# An Urgent Need for a Common Framework for the Articulation, Design and Reporting of Surgical System Strengthening Interventions: Comment on "Improving Access to Surgery Through Surgical Team Mentoring – Policy Lessons From Group Model Building With Local Stakeholders in Malawi"

**DOI:** 10.34172/ijhpm.2022.6993

**Published:** 2022-04-27

**Authors:** Joseph S. Hanna

**Affiliations:** ^1^Robert Wood Johnson Medical School, Rutgers University, New Brunswick, NJ, USA.; ^2^Rutgers Global Health Institute, Rutgers University, New Brunswick, NJ, USA.

**Keywords:** Global Surgery, Surgical System Strengthening, Malawi

## Abstract

Nearly 60% of the world’s inhabitants lack access to timely, safe, and ffordable emergency and essential surgical, anesthetic, and obstetric (SAO) services when needed. Although acknowledged as an important step in resolving this disparity, situation analysis informed development of national surgical, obstetric and anesthesia plans (NSOAPs) has not been performed widely. There are even fewer published examples of NSOAP driven SAO system vulnerability resolving policy interventions, potentially hindering broader acceptance and drafting. Thus, there is urgent need for alignment of academic global surgery activities through a common framework for SAO strengthening intervention articulation, design and reporting which can be informed by the Malawian experience and others. This is a logical next step in the evolution of surgical system science as we move towards the articulation of actionable inequity resolving interventions through stakeholder engagement embedded in a plan-do-study-act (PDSA) model for iterative refinement of strengthening policies.

I read with great interest the article entitled “Improving Access to Surgery Through Surgical Team Mentoring – Policy Lessons From Group Model Building With Local Stakeholders in Malawi” and commend Broekhuizen and colleagues for the development, implementation and analysis of Malawi’s workforce strengthening pilot.^1^ Since 2008, a robust body of evidence has exposed the staggering lack of access experienced by a majority of global inhabitants to timely, safe and affordable emergency and essential surgical care when needed.^2^ A new context for surgical systems analysis and strengthening was brought about by the landmark Lancet publication Global Surgery 2030, and the concomitant prioritization of surgery, anesthesia, and obstetric (SAO) advocacy and policy by the World Health Organization (WHO).^2^ Initially proposed by the Lancet Commission on Global Surgery (LCoGS) in 2015, six core surgical indicators were identified to define, assess, and inform surgical system preparedness, service delivery, and financial risk protection. Description of the LCoGS core indicators established a common global language with which surgical system situation analysis could be objectively and consistently performed. By design, this construct aligns situation analysis with the WHO health system building blocks to inform development of national surgical, obstetric and anesthesia plans (NSOAP).^3^ This framework offers a structured, evidence-based platform upon which policy may be elaborated, and therefore financial investment made in surgical system strengthening by policy makers. Despite broad acknowledgement of this framework, as evidenced in part by the call to action in World Health Assembly Resolution 68.15 and addition of the LCoGS indicators to the World Development Indicator dataset, few nations have drafted an NSOAP based on an LCoGS core indicator based situation analysis.^4^ Even fewer have successfully developed NSOAP driven interventional policies to address SAO system vulnerabilities.^5^ Therefore, development of a framework to guide progression from a national LCoGS indicator based situation analysis to the articulation of specific, measurable, achievable, realistic and timely (SMART) targets achieved through an iterative plan-do-study-act (PDSA) cycle based implementation to resolve this global crisis is imperative.^6,7^

## Malawian Experience

Following completion of a surgical system situation analysis in Malawi funded by the SURG-Africa project, Broekhuizen and colleagues set out to increase district level surgical capacity through task shifting using local resources.^1^ To achieve, this, a mentoring program was proposed whereby specialist providers from a central hospital (Bellwether capable tertiary care center) provided mentorship to district hospital non-specialist providers (clinical officers) who face numerous challenges to providing higher complexity care including training gaps, supply shortages, financial constraints and insufficient infrastructure. Leveraging a participatory action approach and group model building exercises, the study team engaged a broad representation of stakeholders to assess the local situation and scale-up needs in Nsanje in the Southern region, and to explore the dynamics of mentoring, sustainability and replicability in Salima in the Central region. These activities informed the development of a surgical team mentorship model which was implemented and evaluated over the course of two years. To inform sustainability and a future national implementation, the authors modeled the resource requirements for various expansion scenarios based on data collected during the study and other projects supported by the SURG-Africa project.

The study team skillfully employed four important concepts to achieve success in their pilot implementation. Together, these begin to lay the foundation for a basic framework for effective identification, design and sharing of SAO system strengthening interventions. The first, is the need for partnership with the ministry of health to achieve central advocacy, coordination, program monitoring and development. These actions are critical for a successful pilot and subsequent nationwide dissemination, implementation, and sustainability. These steps are explored in the United Nations Institute for Training and Research National Surgical, Obstetric and Anesthesia Planning manual.^8^ Second is the central role of participatory action throughout the development of SAO system strengthening interventions. As previously described, the stakeholder as leader informs identification of an intervention to achieve social change that is context and resource sensitive, culturally prioritized and sustainable.^9^ Third, based on their previously performed granular situation analysis which identified unnecessary transfers to central hospitals as one cause of insufficient local access to SAO services, the study team intuitively articulated a SMART objective. Provision of mentorship to empower clinical officers to undertake a wider range of procedures was proposed as a mechanism to build local capacity. Successfully achieving funding of an intervention through policy is in large part dependent on defining a SMART objective with clear description of these five characteristics.^6,10^ Fourth, the authors ensured program flexibility to incorporate “articulations, workarounds and muddling through that keep the show on the road”, in other words, an iterative cycle of study and action for implementation improvement. Overall, the pilot description can be reformulated as an intervention that incorporates planning and situation analysis to identify a SMART objective, doing (ie, the intervention), study (ie, assessment of the intervention effectiveness) and action for the next iteration (PDSA).^7^ The PDSA cycle is a model for rigorous iterative improvement that has been recognized by organizations such as the Institute for Healthcare Improvement as a robust tool for the implementation of breakthrough interventions. Notably, the study phase of PDSA promotes objective assessment of the outcome to inform intervention strengths and weaknesses which is then followed by action to improve the implementation.^9^ This is exemplified by the reported cost impact analysis which demonstrated a differential impact on district hospitals that was directly proportional to increasing distance from the nearest central hospital. This exposes a critical opportunity to ensure future success of a national scale-up by ensuring proactive management of material resources to prevent facility collapse due to unrecouped cost losses.

## Enhancing Strengthening Initiatives

Notwithstanding the strengths of the present study, there are opportunities to build upon the four concepts exemplified in this work for the development of a common framework. First, a detailed description of the pilot clinical intervention, patient population, and quality outcome is essential to inform broader applicability. The present study is based on a task-shifting model previously described by the COST-Africa project wherein education is provided by specialist provider mentors to clinical officer mentees to enable performance of basic surgical and obstetric care in local district hospitals.^11^ The viability of task-shifting in healthcare is influenced by a myriad of variables such as local laws and regulations, human resource availability, education opportunities, career development opportunities and culturally accepted norms.^12^ However, the value of task-shifting remains unclear as the clinical outcomes of such initiatives have not been rigorously characterized.^2^ While the task-shifting model in the present study is based on the COST-Africa project which described positive results, the absence of procedure specific risk stratification incorporating disease process, acuity, patient age and comorbidities when comparing the outcomes between clinical officers and specialist providers is a critical limitation.^11^ Furthermore, the risk profile of patients treated by clinical officers vs. specialist providers is undescribed, raising questions about patient selection bias which may be influenced by factors such as provider experience or confidence, ultimately affecting observed clinical outcomes. While evaluation of surgical quality in the course of such implementations may seem like a daunting task, the consequences of inadvertently delivering poor quality care are immense and cannot be overlooked.^13^ To that end, McCrum and colleagues have described a program for the successful implementation of a quality improvement program in low resource settings.^14^ These and other characteristics of task-shifting models are critical factors to the decision-making process for a community or nation that may be considering a local implementation, and therefore should be clearly and objectively reported.

Second, ongoing education and advocacy for needed investment to achieve SAO system strengthening is vital. The economic cost of inaction is estimated to be many fold higher than the financial investment needed to close the local and global access gap to emergency and essential SAO services.^2,15^ Furthermore, strengthening without investment, or worse through unintended cost-shifting, is not sustainable and as demonstrated in the present work, may result in great harm to local communities at risk of losing under-resourced safety net health facilities. Effective advocacy for financial investment may be best achieved by demonstrating return on investment through a value-based justification as exemplified by the economic modeling performed by Meara and colleagues.^2^ Therefore, when possible, a proposed financial investment should be reported in the context of value as the quotient of quality and cost for two reasons. The first is to ensure linkage of the quality outcome of any proposed clinical intervention with the cost proposal to ensure appropriate investment in interventions effective in strengthening the delivery of safe SAO services. Second, an evaluation of value is important to inform intervention prioritization as limited human and material resources are apportioned.

 Based on the performance of the presented pilot mentorship program, the authors predict that a nation-wide implementation could be a cost-effective intervention to improve local access to emergency and essential SAO services. Notwithstanding this modeled benefit, the authors retrospectively identified several vulnerabilities to local sustainability and a broader, national dissemination and implementation. First, given the potential cost impact to distant rural district hospitals, without national investment, these safety net facilities are at risk of collapse due to the impact of cost-shifting. Second, vulnerabilities recognized within the mentor-mentee relationship included instability due to frequent clinical officer re-assignment, and unclear articulation and recognition of program benefits to mentors and mentees. Finally, the importance of investment in future gain with a potentially extended time horizon when viewed through a local resource constrained lens was recognized as a significant barrier to continuation of the program at the local level and for national expansion. The transparent identification of these vulnerabilities highlights the importance of embedding SMART objective(s) within a PDSA cycle for iterative vulnerability resolution and intervention strengthening to achieve ultimate national success.

## Conclusion

I congratulate Broekhuizen on a well-designed and impactful intervention to strengthen the SAO workforce in rural Malawi for improved local access to emergency and essential SAO services. While the face validity of an LCoGS core surgical indicator based situation analysis and NSOAP process is strong, the present work and recent report from Binda and colleagues demonstrate the significant challenge in moving from a national situation analysis to planning and ultimately specific policy driven intervention implementation. In their mentoring program pilot, Broekhuizen and colleagues intuitively leveraged several key steps necessary for a successful intervention implementation. Identifying these steps and reframing them in terms of design, implementation and reporting concepts commonly used in public health enables alignment of SAO system strengthening program development and description with language used by the broader community. By expanding on this list with the inclusion of consistent and objective reporting of healthcare outcomes and description of proposed value-based funding mechanisms, we can build a common framework for sharing successful intervention strategies. Ultimately, by aligning academic global surgery system strengthening interventions through a proposed common framework for articulation, design and reporting (Box 1), we can strengthen global advocacy, collaboration, and success. This is a logical next step in the evolution of surgical system science as we move together towards the articulation of SMART actionable gap resolving interventions through a participatory action enhanced stakeholder engagement embedded in a PDSA model for iterative refinement of strengthening policies.

**Box 1. Proposed Global Surgery Disparity Resolution Intervention Articulation, Design, and Reporting Framework**

- Development of partnership with national executive sponsor (eg, Ministry of Health)
- Participatory action informed development of SAO system strengthening interventions
- Completion of a granular situation analysis to inform identification of precise SAO system vulnerabilities for the articulation of a SMART objective informed intervention
- Development of a pilot intervention embedded in a PDSA cycle informed implementation design
- Detailed reporting of the clinical intervention, clinical quality outcomes, and cost impact
- Exploration of a value-based funding mechanism for intervention implementation

----------------

Abbreviations: SAO, surgery, anesthesia, and obstetric; PDSA, plan-do-study-act; SMART, specific, measurable, achievable, realistic and timely.

## Ethical issues

 Not applicable.

## Competing interests

 Author declares that he has no competing interests.

## Author’s contribution

 JSH is the single author of the paper.
